# Agreement between self-reported morbidity and pharmacy claims data for prescribed medications in an older community based population

**DOI:** 10.1186/s12877-020-01684-8

**Published:** 2020-08-10

**Authors:** Clionadh Mannion, John Hughes, Frank Moriarty, Kathleen Bennett, Caitriona Cahir

**Affiliations:** 1 Department of Pharmacology and Therapeutics, University of Dublin, Trinity College Dublin, Dublin, Ireland; 2 Division of Population Health Sciences, Royal College of Surgeons in Ireland, Dublin 2, Ireland; 3 Health Research Board Centre for Primary Care Research, Royal College of Surgeons in Ireland, Dublin, Ireland; 4 The Irish Longitudinal Study on Ageing, Trinity College Dublin, Dublin, Ireland

**Keywords:** Agreement, Self-report, Rx-risk, Rx-risk-V, Morbidity, Polypharmacy, Older people

## Abstract

**Background:**

Studies have indicated variability around prevalence estimates of multimorbidity due to poor consensus regarding its definition and measurement. Medication-based measures of morbidity may be valuable resources in the primary-care setting where access to medical data can be limited. We compare the agreement between patient self-reported and medication-based morbidity; and examine potential patient-level predictors of discordance between these two measures of morbidity in an older (≥ 50 years) community-based population.

**Methods:**

A retrospective cohort study was performed using national pharmacy claims data linked to The Irish LongituDinal study on Ageing (TILDA). Morbidity was measured by patient self-report (TILDA) and two medication-based measures, the Rx-Risk (< 65 years) and Rx-Risk-V (≥65 years), which classify drug claims into chronic disease classes. The kappa statistic measured agreement between self-reported and medication-based morbidity at the individual patient-level. Multivariate logistic regression was used to examine patient-level characteristics associated with discordance between measures of morbidity.

**Results:**

Two thousand nine hundred twenty-five patients were included (< 65 years: *N* = 1095, 37.44%; and ≥ 65 years: *N* = 1830 62.56%). Hypertension and high cholesterol were the most prevalent self-reported morbidities in both age cohorts. Agreement was good or very good (κ = 0.61–0.81) for diabetes, osteoporosis and glaucoma; and moderate for high cholesterol, asthma, Parkinson’s and angina (κ = 0.44–0.56). All other conditions had fair or poor agreement. Age, gender, marital status, education, poor-delayed recall, depression and polypharmacy were significantly associated with discordance between morbidity measures.

**Conclusions:**

Most conditions achieved only moderate or fair agreement between self-reported and medication-based morbidity. In order to improve the accuracy in prevalence estimates of multimorbidity, multiple measures of multimorbidity may be necessary. Future research should update the current Rx-Risk algorithms in-line with current treatment guidelines, and re-assess the feasibility of using these indices alone, or in combination with other methods, to yield more accurate estimates of multimorbidity.

## Key points

### Key findings and implications

1. Agreement between patient self-reported morbidity and medication-based measures of morbidity (Rx-Risk and Rx-Risk-V) was mainly moderate or fair. Diabetes was the only condition for which the level of agreement was found to be very good.
2. The results of our study indicate that neither measure of morbidity is completely reliable, and we suggest that researchers may require multiple measures (self-report and medication-based measures of morbidity) to fully capture accurate prevalence estimates of multimorbidity.
3. Our study identified several limitations of the current versions of the Rx-Risk indices, which require updating if medication-based measures of morbidity are to be used to assess the epidemiology of chronic conditions and multimorbidity.

## Background

Multimorbidity is commonly defined as the presence of two or more chronic medical conditions and its prevalence has been shown to increase with age [1]. As the world’s older population continues to grow, multimorbidity has become an important public health issue capturing the attention of researchers, healthcare professionals, as well as policy makers. Indeed, for healthcare systems to effectively adapt and manage the delivery of healthcare to our growing older population, an accurate description of the epidemiology of chronic conditions is required. However, to date, studies in the literature reveal wide disparities in prevalence estimates of multimorbidity, ranging from 3.5 to 95.1% [2, 3]. This large variability is thought to be due to the lack of standards defining multimorbidity and validated methods for how it should be measured [4]. A recent systematic review reported 132 definitions of multimorbidity involving 1631 different criteria [5]. In addition, the appropriateness of different measures of multimorbidity is also variable depending on both the outcome of interest as well as the type of data that is available [6].

Measures of multimorbidity include diagnosis-based measures (e.g. Charlson Index) based on hospital diagnosis codes (ICD codes), [7] medication-based measures (e.g. Rx-Risk, and Rx-Risk-V for those aged ≥65 years) based on pharmacy data, [8] and patient self-report. Diagnosis-based measures of multimorbidity are the most common measures and are generally based on hospital or physician records [6]. Medication-based measures of multimorbidity include the Rx-Risk and Rx-Risk-V – two algorithms which determine an individual’s current comorbidities based on their dispensed medication. The Rx-Risk indexes only include morbidities for which a medicine could be prescribed and include 42 categories of morbidities based on the World Health Organisation (WHO) Anatomical Therapeutic Classification (ATC) system [9–11]. The Rx-Risk and Rx-Risk-V have good reliability and criterion validity against ICD-9 diagnoses and have been shown to predict costs of care, mortality, and health care utilisation [12]. Previous studies have reported medication-based measures of morbidity (such as the Medicines Disease Burden Index (MDBI) and Rx-Risk-V) to be useful in epidemiological studies when adjusting for comorbidity [13]. However, there are few studies describing the use of these indices to directly measure chronic conditions. Patient self-report is also a valid method of identifying disease categories. A study of older patients with multimorbidity reported good agreement between patient self-report and general practitioner (GP) report for a wide range of diseases [14].

A number of studies have compared the different measures of multimorbidity with differing results [6, 15, 16]. A study of older primary care patients in Ireland found that medication-based measures of multimorbidity such as Rx-Risk-V performed better than diagnosis-based measures of multimorbidity in predicting emergency and ambulatory care sensitive (ACS) admissions [17]. Studies comparing patient self-report and diagnosis-based measures of multimorbidity have reported a stronger association between self-report measures of multimorbidity and quality of life and functional outcomes than diagnosis-based measures [18, 19]. However, no previous research has compared self-reported morbidity in the primary care or community setting with the Rx-Risk measures of morbidity. Comparison between self-reported morbidity data and pharmacy records is important in order to understand the relative merits of each measure of morbidity and the potential for misclassification, particularly in the community setting where access to medical or clinical data can be limited.

Studies have also indicated that agreement between self-report measures and other measures of morbidity might be influenced by patient recall bias [20]. Patient recall has been reported to be influenced by age, marital status and education [21]. There is also some evidence that cognition and memory influence patient recall [22]. The impact of these factors needs to be explored further when assessing and comparing measures of morbidity. The aim of this study was to: (1) compare the agreement between patient self-reported morbidity and medication-based morbidity (Rx-Risk and Rx-Risk-V); and (2) examine potential patient-level predictors of discordance between the two measures of morbidity; including demographic, cognitive, and mental health factors in an older community based population.

## Methods

The STrengthening the Reporting of Observational Studies in Epidemiology (STROBE) guidelines were used in the reporting of this study [23].

### Study population

This was a retrospective cohort study using data from a national pharmacy claims database, the Health Service Executive-Primary Care Reimbursement Service (HSE-PCRS) General Medical Services (GMS) scheme, linked to the first wave of The Irish LongituDinal study on Ageing (TILDA). TILDA is a nationally representative sample of community dwelling individuals aged ≥50 years in Ireland. The sampling framework is based on the Irish Geodirectory, a comprehensive and up-to-date listing and mapping of residential addresses in Ireland compiled by the Ordinance Survey Office; and participants aged ≥50 years were randomly selected using the RANSAM sampling procedure. This meant that each residential address in Ireland had an equal probability of selection, and thus ensured that the TILDA sample was representative of the Irish population aged ≥50 years. The first wave of data collection began in October 2009 through to February 2011 (*N* = 8175 participants aged ≥50 years), where participants completed a computer-aided personal interview (CAPI) and a health assessment measuring their health, economic and social circumstances. Further information on TILDA’s study design and sampling framework is described in detail elsewhere [24].

The HSE-PCRS GMS scheme is the largest pharmacy claims dataset in Ireland, covering more than 40% of the general Irish population [25]. It is means tested and provides free health services, including medications, to eligible persons in Ireland. Qualification for the GMS scheme is on the basis of income related means-testing. Automatic entitlement for those aged ≥70 years occurred between July 2001 and December 2008; however, since January 2009 (current study period), means-testing was introduced, but with a higher income threshold than the general population. As of 2013, 90% of men and 94% of women in the general population aged ≥70 years were eligible [26]. The HSE-PCRS GMS pharmacy claims data were available for consenting TILDA participants aged ≥50 years with GMS eligibility (*N* = 2925).

Within the HSE-PCRS-GMS pharmacy claims data, prescriptions are coded using the WHO ATC classification system; and prescriber information, defined daily doses, strength, quantity, method and unit of administration of each drug dispensed are all available. Pharmacy claims data was extracted for 1 year prior to each participant’s TILDA interview. GMS patients typically receive their medications on a monthly basis [27].

### Self-reported morbidity

As part of the TILDA interview, participants were asked to report if they had any of the following doctor-diagnosed chronic diseases: high blood pressure or hypertension; high cholesterol; angina; congestive heart failure; heart attack; diabetes; stroke or mini-stroke; abnormal heart rhythm; arthritis; osteoporosis; cancer; Parkinson’s disease; emotional nervous or psychiatric problems; alcohol or substance abuse; dementia; serious memory impairment; stomach ulcers; glaucoma; incontinence; or chronic pain. Participants were also asked to self-report urinary incontinence in the past 12 months, as well as pain (moderate or severe), and if they were taking medication for pain management. If participants reported that they had arthritis, they were asked to clarify the type of arthritis (e.g. osteoarthritis, rheumatoid arthritis, some other kind of arthritis). Similarly, if participants reported emotional, nervous or psychiatric problems, they were asked to clarify from a list of conditions (e.g., anxiety, depression, emotional problems, psychosis, manic depression).

### Medication-based measures of morbidity – Rx-risk and Rx-risk-V

The Rx-Risk and Rx-Risk-V indices were applied to the HSE-PCRS pharmacy claims data. The Rx-Risk index was applied to the population aged < 65 years, while the Rx-Risk-V was applied to the population aged ≥65 years. The Rx-Risk and Rx-Risk-V are algorithms that classify prescription drug fills into chronic disease classes for older populations based on the WHO ATC classification system [9–11]. Within the Rx-Risk-V, cardiac disease is separated into a number of categories: anticoagulation; antiplatelet agents; arrhythmias; congestive heart failure (CHF)/hypertension; hypertension; ischaemic heart disease (IHD)/angina; and ischaemic heart disease (IHD)/hypertension [9]. For a medication to be eligible as a measure of morbidity per Rx-Risk and Rx-Risk-V chronic disease classes, a patient was required to have been dispensed two or more consecutive prescriptions of the medication in question (e.g., ‘donepezil’ was required to be dispensed on ≥2 consecutive prescriptions to link this medication with the Rx-Risk-V condition ‘dementia’). This definition has previously been used by other pharmacoepidemiological studies [28].

### Comparison of self-reported morbidity with Rx-risk and Rx-risk-V

Each self-reported condition in TILDA was matched to the equivalent Rx-Risk and Rx-Risk-V condition at the individual patient level for those aged < 65 years and ≥ 65 years, respectively. This was performed by consensus between two pharmacists (FM & CM). For some self-reported conditions, the ATC classes of medications were not specific to these conditions – e.g. anti-thrombotic agents (B01AC04 – B01AC30) were matched to the self-reported condition of a heart attack and also to stroke. There were four self-reported TILDA conditions which could not be matched to an Rx-Risk or Rx-Risk-V condition, but the prevalence was low (Appendix 1 in Tables 5, 6 and 7). The Rx-Risk and Rx-Risk-V also reported conditions which patients had not been asked about during their TILDA interview. (Appendix 1 in Tables 5, 6 and 7).

### Patient-level characteristics associated with discordance between the two measures of morbidity

Patient characteristics were assessed to determine discordance (patient recall bias) between self-reported morbidity (TILDA) and the Rx-Risk (< 65 years) and Rx-Risk-V (≥ 65 years) medication-based measures of morbidity. These characteristics were age, gender, marital status, education, poor delayed recall, depression and polypharmacy. Marital status was sub-categorised into married, never married, separated or divorced. Education was categorised into primary/none, secondary or third/higher level education. Delayed recall, based on participants being presented with 10 words during the interview and being later asked to recall as many as possible, was defined as poor where 3 or fewer words were recalled. Depression was defined as scoring 16 or greater on the Centre for Epidemiologic Studies Depression Scale (CES-D) [29]. Polypharmacy was defined as reporting regular use of five or more prescription medications [30].

### Statistical methods

Agreement between self-reported morbidity (TILDA) and the Rx-Risk and Rx-Risk-V measures of morbidity (pharmacy claims) was assessed using Cohen’s Kappa statistic, as neither source was considered to be a gold standard for reporting morbidity. Interpretation of the value of *Kappa* was as follows: poor (< 0.20), fair (0.20–0.40), moderate (0.41–0.60), good (0.61–0.80), and very good (0.81–1.00) [31].

Multivariate logistic regression was used to examine the association between the patient-level characteristics and discordance between the two measures of morbidity. Adjusted odds ratios (OR) and 95% confidence intervals (CI) are presented. Discordance was defined as participants reporting to have the condition in the absence of any dispensed medication for the condition, per Rx-Risk (< 65 years) or per Rx-Risk-V (≥ 65 years), and participants reporting to not have the condition, but medication was found to be dispensed for the condition, per Rx-Risk (< 65 years) or Rx-Risk-V (≥ 65 years). All significance tests were two-tailed. Statistical significance was set at *P* < 0.05, after adjustment for a false discovery rate of 5% [32]. Analyses were performed using Stata SE Version 14.0 statistical package (StataCorp, College Station, TX).

## Results

### Study population

In total, 2925 patients were included in this cohort study: 1095 (37.44%) patients were aged < 65 years and 1830 (62.56%) were aged ≥65 years. Characteristics of the study participants are presented in Table 1. On average patients aged < 65 years had 3 (SD 2.17) conditions per the Rx-Risk and patients aged ≥65 years had 6 (SD 3.27) conditions per the Rx-Risk-V. The proportion of patients with third/higher level education was relatively low across both age groups (< 65 years: *N* = 175 (15.98%), ≥ 65 years: *N* = 317 (17.32%)). Poor delayed recall (< 65 years: *N* = 177 (16.16%), ≥ 65 years: *N* = 560 (30.60%)) and polypharmacy (< 65 years: *N* = 301 (27.49%), ≥ 65 years: *N* = 733 (40.05%)) were significantly more prevalent in the older cohort compared to the younger cohort (*p* < 0.05).

**Table 1:** Characteristics of study participants by age <  65 years and ≥ 65 years

	< 65 years (***N*** = 1095)	≥ 65 years (***N*** = 1830)
**Age**	56.22 (55.9–56.54)	73.9 (73.67–74.2)
**Gender**		
Male	417 (38.08)	839 (45.85)
Female	678 (61.92)	991 (54.15)
**Marital Status**		
Married	705 (64.38)	991 (54.15)
Never Married	126 (11.51)	189 (10.33)
Separated/Divorced	163 (14.89)	63 (3.44)
Widowed	101 (9.22)	587 (32.08)
**Education**		
Primary/none	431 (39.36)	941 (51.42)
Secondary	489 (44.66)	570 (31.15)
Third/Higher Level	175 (15.98)	317 (17.32)
**Poor delayed recall (Yes)**	177 (16.16)	560 (30.60)
**Depression (Yes)**	262 (23.93)	210 (11.48)
**Polypharmacy (Yes)**	301 (27.49)	733 (40.05)

Data presented as N (%) or mean (CI), unless otherwise stated

### Agreement between self-reported morbidity and medication-based measures of morbidity (Rx-risk and Rx-risk-V)

Tables 2 and 3 present a comparison between the number and percentage of patients’ self-reported morbidities compared to the Rx-Risk (Table 2: aged < 65 years) and Rx-Risk-V (Table 3: aged ≥65 years) measures of morbidity. High blood pressure or hypertension (< 65 years: *N* = 438 (40.00%), ≥ 65 years: *N* = 964 (52.68%)) and high cholesterol (< 65 years: *N* = 458 (41.83%), ≥ 65 years: *N* = 733 (40.05%)) were the most prevalent self-reported morbidities in both age cohorts in the TILDA dataset. High cholesterol was also found to be highly prevalent in the Rx-Risk (*N* = 454, 41.46%) and Rx-Risk-V (*N* = 1002, 54.75%) measures of morbidity. Other prevalent Rx-Risk and Rx-Risk-V conditions included arthritis (Rx-Risk: *N* = 688 (62.83%)), stomach ulcers (Rx-Risk: *N* = 518 (47.31%), Rx-Risk-V: *N* = 947 (51.75%)), stroke (Rx-Risk-V: *N* = 952 (52.02%)), heart attack (Rx-Risk-V: *N* = 952 (52.02%)) and other heart trouble (Rx-Risk-V: *N* = 884 (48.31%)).

**Table 2:** Agreement (kappa statistic and standard error) between self-reported morbidity in TILDA and Rx-Risk algorithm (< 65 years)

TILDA			Rx-Risk Pharmacy Claims			Kappa ***(κ)***	Standard Error
Self-reported morbidity	N	%	Medication-based Morbidity (***ATC***)	N	%		
Diabetes or high blood sugar	110	(10.05)	Diabetes *(A10AB01-A10BG03, A10BH, A10BX)*	98	(8.95)	0.81	0.03
Glaucoma	25	(2.28)	Glaucoma *(S01EA01-S01EB03, S01EC03-S01EX)*	19	(1.74)	0.58	0.03
High Cholesterol	458	(41.83)	Hyperlipidaemia *(C10AA01-C10BX17)*	454	(41.46)	0.52	0.03
Asthma	133	(12.15)	Asthma *(R03AA-R03AL, R03BA-R03BX, R03CA-R03CC, R03DA-R03DX)*	278	(25.39)	0.47	0.03
High blood pressure or Hypertension	438	(40.00)	Hypertension *(C03AA01-C03BA11, C03DA01-C03EA01, C09BA02-C09BA09, C09DA01-C09DA07, C02AB01-C02AC05, C02DB02-C02KX01)*	190	(17.35)	0.40	0.03
Cancer or a malignant tumour	80	(7.31)	Malignancies *(L01AA01-L01XX31)*	23	(2.10)	0.31	0.02
Depression	129	(11.78)	Depression *(N06AA01-N06AG02, N06AX)*	282	(25.75)	0.24	0.03
Stroke (cerebral vascular disease)	78	(7.12)	Anti-platelet therapy *(B01AC04-B01AC30)*	291	(26.58)	0.23	0.02
Parkinson	3	(0.27)	Parkinson’s disease *(N04AA01-N04BX02)*	15	(1.37)	0.22	0.02
Heart attack (including myocardial infarction or coronary thrombosis)	54	(4.93)	Anti-platelet therapy *(B01AC04-B01AC30)*	291	(26.58)	0.21	0.02
Manic depression	12	(1.10)	Bipolar disorder *(N05AN01)*	12	(1.10)	0.16	0.03
Emotional nervous or psychiatric problem, such as depression or anxiety	180	(16.44)	Anxiety *(N05BA01-N05BA12)*	252	(23.01)	0.15	0.03
Anxiety	101	(9.22)	Anxiety *(N05BA01-N05BA12)*	252	(23.01)	0.14	0.03
Cirrhosis or serious liver damage	13	(1.19)	Liver disease *(A05AA01-A05BA08, J05AF05, J05AF07, J05AF11)*	1	(0.09)	0.14	0.02
Stomach ulcers	119	(10.87)	GORD & Peptic ulcer *(A02B, A02BB, A02BC)*	518	(47.31)	0.13	0.02
Arthritis (including osteoarthritis or rheumatism)	310	(28.31)	Rheumatoid Arthritis *(M01AA-M01CX, M02AA-M02AX, L01BA01, L04AB01-L04AB05, L04AD01, L04AX03)*	688	(62.83)	0.13	0.02
Any other heart trouble	49	(4.47)	Ischaemic heart disease/hypertension *(C07AA01-C07FB07, C08CA01-C08DB01)*	303	(27.67)	0.09	0.02
Rheumatoid arthritis only	119	(10.87)	Rheumatoid Arthritis *(M01AA-M01CX, M02AA-M02AX, L01BA01, L04AB01-L04AB05, L04AD01, L04AX03)*	688	(62.83)	0.06	0.02
Mini-stroke or TIA	33	(3.01)	Anti-platelet, Anti-coagulation therapy^**a**^ *(B01AC04-B01AC30, B01AA03-B01AB06)*	335	(30.6)	0.05	0.01

*ATC* Anatomical Therapeutic Chemical

*GORD* Gastro-Oesophageal Reflux Disease

^a^Anti-coagulant counted if patient co-prescribed antiarrhythmic for Atrial Fibrillation (i.e., if patient not in sinus rhythm) [33]

**Table 3:** Agreement (kappa statistic and standard error) between self-reported morbidity in TILDA and Rx-Risk-V algorithm (≥ 65 years)

TILDA			Rx-Risk-V Pharmacy claims			Kappa ***(κ)***	Standard Error
Self-reported morbidity	N	%	Medication-based Morbidity (***ATC***)	N	%		
Diabetes or high blood sugar	222	(12.13)	Diabetes *(A10AB01-A10BG03, A10BH, A10BX)*	191	(10.44)	0.81	0.02
Glaucoma	82	(4.48)	Glaucoma *(S01EA01-S01EB03, S01EC03-S01EX)*	104	(5.68)	0.69	0.02
Osteoporosis	253	(13.83)	Osteoporosis/Paget’s disease *(M05BA01-M05BB09, M05BX03, G03XC01, A12AX92)*	306	(16.72)	0.61	0.02
Parkinson	17	(0.93)	Parkinson’s disease *(N04AA01-N04BX02)*	40	(2.19)	0.56	0.02
Angina	213	(11.64)	Angina *(C01DA02-C01DA14, C01DX16, C01EB17-C01EB18)*	194	(10.60)	0.51	0.02
High Cholesterol	733	(40.05)	Hyperlipidaemia *(C10AA01-C10BX17)*	1002	(54.75)	0.44	0.02
Manic depression	5	(0.27)	Bipolar disorder *(N05AN01)*	6	(0.33)	0.36	0.02
High blood pressure or Hypertension	964	(52.68)	Hypertension *(C03AA01-C03BA11, C03DA01-C03EA01, C09BA02-C09BA09, C09DA01-C09DA09, C02AB01-C02AC05, C02DB02-C02KX01)*	591	(32.30)	0.36	0.02
Pain (taking pain medication)	464	(25.36)	Pain (Opioids) (N02AA01-N02AX02) Pain (Inflammation) (M01AB01, M01AH06)	523	(28.58)	0.35	0.02
Pain	726	(39.67)	Pain (Opioids) (N02AA01-N02AX02) Pain (Inflammation) (M01AB01, M01AH06)	523	(28.58)	0.27	0.02
Abnormal Heart Rhythm	221	(12.08)	Arrhythmia *(C01AA05, C01BA01-C01BD01, C01BD07)*	103	(5.63)	0.26	0.02
Depression	78	(4.26)	Depression (*N06AA01-N06AG02, N06AX*)	359	(19.62)	0.21	0.02
Dementia	5	(0.27)	Dementia *(N06DA02, N06DA01)*	24	(1.31)	0.20	0.02
Chronic lung disease such as chronic bronchitis or emphysema	97	(5.30)	Chronic airways disease *(R03AC02-R03DC03)*	457	(24.97)	0.19	0.02
Cancer or a malignant tumour	177	(9.67)	Malignancies *(L01AA01-L01XX31)*	62	(3.39)	0.19	0.02
Emotional nervous or psychiatric problem, such as depression or anxiety	115	(6.28)	Anxiety *(N05BA01- N05BA12)*	327	(17.87)	0.15	0.02
Congestive heart failure	31	(1.69)	Chronic heart failure *(C03CA01-C03CC01, C09AA01-C09AA10, C09CA01, C09CA03, C09CA06-C09CA07)*	269	(14.70)	0.13	0.01
Cirrhosis or serious liver damage	10	(0.55)	Liver disease *(A05AA01-A05BA08, J05AF05, J05AF07, J05AF11)*	6	(0.33)	0.12	0.02
Heart attack (including myocardial infarction or coronary thrombosis)	171	(9.34)	Anti-platelet therapy *(B01AC04-B01AC30)*	952	(52.02)	0.11	0.01
Anxiety	66	(3.61)	Anxiety *(N05BA01-N05BA12)*	327	(17.87)	0.11	0.02
Stomach ulcers	127	(6.94)	GORD & Peptic ulcer *(A02BA, A02BC)*	947	(51.75)	0.10	0.01
Alcohol or substance abuse	28	(1.53)	Alcohol dependence *(N07BB01, N07BB04)*	1	(0.05)	0.07	0.01
Any other heart trouble	101	(5.52)	Ischaemic heart disease/hypertension *(C07AA01-C07FB07, C08CA01-C08DB01)*	884	(48.31)	0.06	0.01
Stroke (cerebral vascular disease)	61	(3.33)	Anti-platelet therapy *(B01AC04-B01AC30)*	952	(52.02)	0.04	0.01
Mini-stroke or TIA	131	(7.16)	Anti-platelet, Anti-coagulation therapy^a^ *(B01AC04-B01AC30, B01AA03-B01AB06, B01AB10)*	1104	(60.32)	0.02	0.01
Incontinence	313	(17.10)	Neurogenic Bladder & Urinary Incontinence *(V07AN)*	17	(0.93)	0.02	0.01
Psychosis	1	(0.05)	Psychotic illness *(N05AA01- N05AX17)*	150	(8.20)	0.01	0.00

*ATC* Anatomical Therapeutic Chemical

*GORD* Gastro-Oesophageal Reflux Disease

^a^Anti-coagulant counted if patient prescribed antiarrhythmic for Atrial Fibrillation (i.e., if patient not in sinus rhythm) [33]

There was very good agreement between the self-reported TILDA measure of diabetes and the Rx-Risk and Rx-Risk-V measures (κ = 0.81). There was also good agreement between self-reported measures of osteoporosis (κ = 0.61) and glaucoma (κ = 0.69) and the Rx-Risk-V measure of these morbidities in the older cohort. Despite the high prevalence of high cholesterol in both measures of morbidity, there was only moderate agreement (κ = 0.52 Rx-Risk, κ = 0.44 Rx-Risk-V) between the two measures. There was moderate agreement also for asthma (κ = 0.47 Rx-Risk), Parkinson’s (κ = 0.56 Rx-Risk-V) and angina (κ = 0.51 Rx-Risk V). Agreement was fair for self-reported high blood pressure or hypertension (Rx-Risk and Rx-Risk-V), heart attack (Rx-Risk), stroke (Rx-Risk), abnormal heart rhythm (Rx-Risk-V), cancer (Rx-Risk), depression (Rx-Risk and Rx-Risk-V) and pain (Rx-Risk-V) and Rx-Risk measures of these conditions (κ = 0.21–0.40). All other conditions had poor agreement (κ = 0.02–0.19); including arthritis (Rx-Risk), chronic lung disease and incontinence (Rx-Risk-V) and emotional, nervous psychiatric problems, anxiety and stomach ulcers (Rx-Risk and Rx-Risk-V) (Tables 2 & 3).

### Patient-level characteristics associated with discordance between the two measures of morbidity

Age, gender, marital status, education, poor delayed recall, depression, and polypharmacy were all associated with discordance between the two measures of morbidity (Table 4). Females were five times more likely to have discordance in reporting osteoporosis (OR = 5.20, 95% Confidence Intervals (CI) 3.33, 8.09, *P* < 0.05). Females were also more likely to have discordance in reporting anxiety (OR = 1.66; 95% CI 1.35, 2.04), emotional problems (OR = 1.57; 95% CI 1.29, 1.92) and depression (OR = 1.46; 95% CI 1.19, 1.79) as well as use of pain medication (OR = 1.43; 95% CI 0.13, 1.80) and incontinence (OR = 2.13; 95% CI 1.59, 2.85). They were less likely to have discordance in reporting stroke and high cholesterol (Table 4).

**Table 4:** Odds ratios (with 95% confidence intervals) for patient-level characteristics associated with discordance between the measures of morbidity (self-report and Rx-Risk and Rx-Risk-V)

	Hypertension	Heart Attack	Stroke	TIA	High Cholesterol	Heart Trouble	Cancer	Emotional Problems	Anxiety
**Age (years)**	1.01 (1.01–1.02)*	1.04 (1.03–1.05)*	1.04 (1.03–1.05)*	1.00 (1.02–1.10)	1.01 (1.00–1.01)	1.04 (1.03–1.05)*	1.02 (1.00–1.03)	0.98 (0.97–0.99)	0.99 (0.98–1.00)
**Gender**									
Male									
Female	0.94 (0.79–1.11)	0.88 (0.74–1.04)	0.62 (0.52–0.74)*	0.60 (0.45–0.80)*	0.68 (0.57–0.81)*	0.92 (0.77–1.09)	0.86 (0.65–1.14)	1.57 (1.29–1.92)*	1.66 (1.35–2.04)*
**Marital Status**									
Married									
Never Married	1.04 (0.79–1.37)	0.69 (0.52–0.92)*	0.69 (0.52–0.92)*	0.88 (0.54–1.42)	0.89 (0.67–1.10)	0.85 (0.65–1.13)	1.00 (0.65–1.55)	0.98 (0.72–1.35)	0.95 (0.69–1.32)
Separated/Divorced	0.97 (0.69–1.35)	0.83 (0.59–1.17)	0.77 (0.55–1.08)	1.34 (0.76–2.36)	0.94 (0.67–1.30)	0.85 (0.61–1.19)	0.79 (0.44–1.40)	1.15 (0.82–1.61)	1.04 (0.73–1.47)
Widowed	1.04 (0.84–1.30)	1.08 (0.86–1.34)	1.11 (0.89–1.38)	1.03 (0.72–1.48)	1.09 (0.87–1.37)	0.91 (0.73–1.14)	0.94 (0.65–1.33)	1.06 (0.82–1.35)	0.94 (0.73–1.22)
**Education**									
Primary/None									
Secondary	0.93 (0.78–1.13)	0.91 (0.83–1.20)	0.95 (0.78–1.15)	0.88 (0.64–1.20)	1.17 (0.97–1.40)	0.99 (0.82–1.19)	1.42 (1.04–1.92)	1.02 (0.83–1.25)	0.97 (0.78–1.20)
Third/Higher Level	0.75 (0.59–0.96)*	1.13 (0.89–1.43)	1.14 (0.90–1.44)	0.78 (0.52–1.17)	1.12 (0.87–1.00)	0.91 (0.72–1.15)	1.37 (0.95–2.00)	0.86 (0.65–1.13)	0.83 (0.63–1.10)
**Poor Delayed Recall (Yes)**	0.97 (0.79–1.17)	1.04 (0.86–1.27)	0.98 (0.81–1.20)	0.75 (0.54–1.04)	0.99 (0.81–1.20)	0.90 (0.75–1.10)	1.00 (0.73–1.37)	1.18 (0.95–1.48)	1.22 (1.62–2.59)
**Depression (Yes)**	0.97 (0.77–1.23)	1.08 (0.85–1.36)	1.03 (0.81–1.30)	1.20 (0.82–1.75)	0.98 (0.77–1.20)	0.92 (0.73–1.16)	1.36 (0.96–1.94)	2.15 (1.71–2.70)*	2.05 (1.62–2.59)*
**Polypharmacy (Yes)**	1.81 (1.52–2.14)*	3.12 (2.64–3.70)*	4.13 (3.47–4.90)*	3.60 (2.69–4.79)*	1.40 (1.18–1.60)*	3.50 (2.97–4.17)*	1.28 (0.97–1.69)	1.16 (0.96–1.41)	1.20 (0.98–1.46)
	Depression only	Stomach ulcers	Asthma	Arthritis (general)	Rheumatoid Arthritis only	Angina	Congestive Heart Failure	Abnormal Heart Rhythm	Lung Disease
**Age**	0.99 (0.98–1.00)	1.01 (1.00–1.02)	1.02 (0.99–1.06)	1.00 (0.98–1.02)	1.00 (0.98–1.03)	1.03 (1.02–1.05)*	1.06 (1.04–1.09)*	1.03 (1.00–1.06)	0.10 (0.97–1.02)
**Gender**									
Male									
Female	1.46 (1.19–1.79)*	1.09 (0.92–1.27)	0.77 (0.54–1.09)	1.02 (0.78–1.32)	1.11(0.86–1.44)	1.13 (0.91–1.4)	1.00 (0.73–1.34)	0.74 (0.54–1.03)	1.17 (0.91–1.50)
**Marital Status**									
Married									
Never Married	1.04 (0.75–1.43)	0.97 (0.75–1.25)	1.10 (0.65–1.87)	0.95 (0.64–1.41)	0.94(0.63–1.39)	1.01 (0.72–1.43)	1.26 (0.76–2.06)	0.64 (0.36–1.13)	1.09 (0.74–1.60)
Separated/Divorced	1.24 (0.88–1.76)	1.01 (0.75–1.37)	1.28 (0.80–2.06)	0.94 (0.66–1.35)	0.79(0.55–1.14)	1.19 (0.68–2.07)	1.43 (0.64–3.21)	0.83 (0.34–2.02)	1.00 (0.53–1.89)
Widowed	1.00 (0.77–1.29)	0.97 (0.79–1.20)	1.65 (0.96–2.82)	0.93 (0.60–1.44)	0.73(0.47–1.13)	0.86 (0.67–1.11)	1.06 (0.74–1.53)	0.88 (0.60–1.27)	0.81 (0.61–1.09)
**Education**									
Primary/None									
Secondary	1.05 (0.85–1.30)	1.12 (0.94–1.33)	0.90 (0.62–1.30)	0.98 (0.74–1.29)	1.04(0.78–1.37)	0.89 (0.71–1.13)	0.75 (0.53–1.06)	1.24 (0.89–1.75)	0.70 (0.53–0.92)
Third/Higher Level	0.80 (0.60–1.07)	0.89 (0.71–1.11)	0.79 (0.48–1.33)	0.70(0.48–1.02)	0.74(0.51–1.07)	0.87 (0.65–1.15)	0.59 (0.37–0.95)	0.99 (0.64–1.54)	0.77 (0.56–1.07)
**Poor Delayed Recall**	1.25 (1.00–1.57)	1.11 (0.93–1.34)	0.76 (0.48–1.22)	1.06 (0.75–1.48)	0.89(0.64–1.25)	0.85 (0.68–1.07)	1.13 (0.82–1.56)	0.93 (0.67–1.30)	1.04 (0.80–1.32)
**Depression (Yes)**	2.34 (1.85–2.95)*	1.34 (1.09–1.67)*	1.30 (0.89–1.91)	1.36 (1.01–1.82)*	1.26(0.94–1.70)	1.00 (0.73–1.38)	1.71 (1.15–2.55)*	1.67 (1.10–2.50)*	1.47 (1.04–1.34)*
**Polypharmacy**	1.60 (1.31–1.95)*	2.17 (1.84–2.55)*	1.82 (1.29–2.58)*	0.79 (0.60–1.04)	1.11 (0.84–1.47)	1.88 (1.50–2.30)*	3.64 (2.67–4.93)*	3.28 (2.40–4.49)*	1.56 (1.23–1.97)*
	Osteoporosis	Psychosis only	Incontinence	Pain	Pain (meds)				
**Age**	1.01 (0.98–1.03)	1.01 (0.98–0.05)	1.02 (0.99–1.04)	0.99 (0.97–1.01)	1.00 (0.98–1.01)				
**Gender**									
Male									
Female	5.20 (3.33–8.09)*	2.00 (1.34–2.97)*	2.13 (1.59–2.85)*	1.20 (0.97–1.50)	1.43 (1.13–1.80)*				
**Marital Status**									
Married									
Never Married	0.76 (0.39–1.45)	1.10 (0.60–2.02)	1.04 (0.65–1.67)	1.19 (0.84–1.67)	1.43 (1.00–2.00)				
Separated/Divorced	0.77 (0.29–2.05)	1.71 (0.76–3.85)	1.37 (0.68–2.70)	1.20 (0.69–2.08)	1.19 (0.66–2.10)				
Widowed	1.07 (0.73–1.56)	0.85 (0.55–1.32)	1.25 (0.91–1.71)	0.94 (0.73–1.22)	0.88 (0.67–1.16)				
**Education**									
Primary/None									
Secondary	1.15 (0.79–1.67)	0.68 (0.44–1.05)	1.10 (0.82–1.49)	0.80 (0.63–1.02)	0.83 (0.64–1.00)				
Third/Higher Level	1.23 (0.76–1.97)	0.89 (0.59–1.59)	1.07 (0.73–1.57)	1.00 (0.75–1.32)	0.78 (0.57–1.00)				
**Poor Delayed Recall**	1.32 (0.92–1.89)	1.40 (0.96–2.06)	1.24 (0.93–1.65)	0.98 (0.78–1.23)	1.17 (0.92–1.40)				
**Depression (Yes)**	1.36 (0.87–2.14)	2.00 (1.27–3.14)*	2.40 (1.70–3.40)*	1.59 (1.16–2.16)*	1.30 (0.93–1.80)				
**Polypharmacy**	1.57 (1.14–2.20)*	1.27 (0.89–1.01)	1.92 (1.48–2.50)*	1.27 (1.03–1.56)*	1.49 (1.19–1.80)*				

Excluded diabetes, Parkinson’s disease, manic depression, cirrhosis, glaucoma, alcohol or substance abuse, and dementia as number of patients misreporting was small (*N* < 120)

* *p* < 0.05

Patients who were never married were less likely to have discordance in reporting a heart attack (OR = 0.69; 95% CI 0.52, 0.92) and stroke (OR = 0.69; 95% CI 0.52, 0.92). Patients with third level education were less likely to have discordance in reporting hypertension (OR = 0.75; 95% CI 0.59, 0.96), compared to those with primary level education (Table 4). Patients with poor delayed recall and depression were more likely to have discordance in reporting anxiety and depression. In general, discordance was higher in patients with polypharmacy (Table 4).

## Discussion

Within a population based study of ageing in Ireland we found that agreement between patient self-reported morbidity and medication-based measures of morbidity (Rx-Risk and Rx-Risk-V), was generally not good; with most conditions achieving only moderate or fair agreement. There was ‘very good’ agreement (κ = 0.81) between self-reported diabetes and pharmacy dispensing records, across both age cohorts. This was the only morbidity common to both age cohorts for which the level of agreement was found to be ‘very good’. Many research studies confirm this same level of agreement for diabetes [22, 34, 35]. This was expected, given that previous research has demonstrated the reliability of reporting to be better in morbidities for which there are clear diagnostic criteria (e.g. diabetes) [36]. Furthermore, with many educational resources promoting self-management of this condition, patients with diabetes are more likely to play an active role in managing their condition (e.g. regular self-monitoring of blood glucose levels; dietary management; recognising and dealing with symptoms, such as hypo- and hyper-glycaemia; and/or medication taking) and are therefore more likely to self-report accurately [37].

There was ‘good’ agreement between both measures of morbidity for osteoporosis and for glaucoma in the older age group. A Multi-Care cohort study of primary care patients in Germany found only moderate agreement between patient-reported and GP-reported osteoporosis [14]. A retrospective cohort study of older patients in a secondary-care setting in Canada, also found moderate agreement for glaucoma between physician and patient reports [38]. Similar to diabetes, patients are required to play an active role in the management of osteoporosis, while glaucoma is very often a comorbidity of diabetes [39].

There was ‘moderate’ agreement between the measures of morbidity for asthma in the younger age cohort (< 65 years). Similar results have been reported for agreement between self-reported asthma and medical record data in older hospitalised patients [40]. There was also ‘moderate’ agreement for high cholesterol in both age cohorts and for angina and Parkinson’s disease in the older age cohort. Other studies have reported lower agreement for high cholesterol and higher agreement for angina and Parkinson’s diseases [14, 41]. Discordance here may be explained by patients managing their cholesterol using non-pharmacological means (e.g., lifestyle modifications, including cardioprotective diet) [42]. Interestingly the prevalence of self-reported angina in TILDA was higher than the prevalence reported by Rx-Risk-V. This may reflect poor patient adherence if prescribed medications were not dispensed.

There was only ‘fair’ agreement between both measures of morbidity for hypertension despite hypertension being the most prevalent self-reported morbidity across both age cohorts. Higher agreement between self-reported anti-hypertensive drug use and pharmacy records, has been reported in a population-based study and a cohort study of older people in the Netherlands [43, 44]. The discordance observed here is likely attributable to the omission of a major group of anti-hypertensives, calcium-channel-blockers (CCBs), in the current version of the Rx-Risk and Rx-Risk-V algorithms [9, 10]. This is significant given that CCBs are recommended as first-line therapy in patients aged > 55 years [45]. Equally, since hypertension is considered to be a condition without symptoms, [46] this may influence patient adherence to anti-hypertensive medications, and their proclivity to fill a prescription for these medications. There was also ‘fair’ agreement for pain in the older age group, with agreement increasing somewhat when self-reported pain specified ‘taking pain medication’. The prevalence of self-reported pain was higher than the medication-based (Rx-Risk-V) prevalence and this may be due to patients managing their pain through non-pharmacological or lifestyle interventions such as physiotherapy and cognitive behavioural therapy [47].

In both age cohorts, there was “poor to fair” agreement between self-reporting of emotional problems (e.g. depression, anxiety) and medication-based measures. These findings are consistent with previous research which found poor agreement between physician diagnosis and patient self-reports of anxiety and depression [48]. This low level of agreement may be due to a potential stigmatisation bias, as only 21.7% (78/359) of patients regularly dispensed anti-depressants self-reported as having depression in the older age cohort [49, 50]. Equally, it may be that certain anti-depressants (e.g. amitriptyline) are being used for other indications, such as neuropathic pain [51, 52].. There was also ‘poor’ agreement in both age cohorts for stomach ulcers, and for incontinence and chronic airways disease (COPD) in the older cohort. Like depression, poor agreement here may be due to gastrointestinal medications being used by patients for other indications, such as preventative or symptomatic reasons [53]. The poor agreement for chronic airways disease may reflect the non-specific question used in TILDA to measure this self-reported morbidity, as there is evidence in the literature that questionnaire design is an important determinant of patient recall. In a US study, the prevalence of self-reported COPD was found to increase when more explicit questions were asked about emphysema, chronic bronchitis and COPD in combination [54]. The poor agreement between the two measures for incontinence is most likely reflective of the current version of the Rx-Risk-V, which compares self-reported urinary incontinence with dispensed ‘diapers and pads (supplies)’. [9].

A number of factors were associated with discordance between the two measures of morbidity; particularly increasing age, poor delayed recall, depression, and polypharmacy. A study determining the agreement between self-reported and diagnosis-based multimorbidity in older community dwelling women reported similar findings, where agreement was found to decrease with decreasing cognition and education, increasing age, and four or more diseases [22]. Multiple chronic conditions and polypharmacy have also been shown to negatively affect cognitive recall [55]. In addition, patients with chronic disease often experience co-morbidities such as mental health conditions and psychosocial difficulties (e.g. depression), and these have been reported to negatively affect self-report [56, 57]; and have been shown to be associated with lower agreement between patient self-reported morbidity and GP report [14]. Discordance per pharmacy claims data may result from poor adherence to prescribed medication, medications being used for other indications, or conditions being managed using non-pharmacological means. Studies have also found that other patient characteristics (e.g., sex) can influence treatment patterns [58]. There is evidence that depression and impaired cognition are predictors of poor adherence and this study found a relationship between poor delayed recall and depression and potential misreporting [59].

### Limitations

This is the first study to assess the reliability of self-reported morbidity by older patients (≥50 years) in a primary care setting in Ireland, against pharmacy dispensing data using two validated measures of morbidity (Rx-Risk and Rx-Risk-V). However, this study has a number of limitations. The study only included patients with a GMS medical card and these patients tend to be older, female, more socially deprived and have reported more chronic diseases, medications and depressive symptoms than the general population [28]. Future studies should compare the agreement between patient self-reported morbidity and medication-based morbidity (Rx-Risk and Rx-Risk-V) in other data sets which include participants from a broader range of socioeconomic and ethnic backgrounds and across different healthcare schemes. It is also not known whether patients adhered to their medications or had discontinued treatment. In addition, the pharmacy claims database used in this study does not include over-the-counter (OTC) items, although this may not be a significant factor as the GMS provides free medical treatment and patients must pay for OTC items [27].

There were also a number of conditions that we were unable to match in both TILDA and the Rx-Risk and the Rx-Risk-V and these were excluded from our study (Appendix 1 in Tables 5, 6 and 7). The two measures of morbidity were compared over a 1 year period but it was not known when participants were actually diagnosed with their self-reported condition(s) or when they initiated medication for their condition. Therefore, there may be differences in agreement for newly diagnosed conditions versus conditions which participants have been managing for a number of years. With this in mind, agreement between pharmacy refill data and self-reported morbidity may vary over time and future research should therefore investigate the relationship between these two measures longitudinally to obtain a clearer understanding of treatment patterns [60]. The statistical significance of the association between the patient-level characteristics and discordance between the two measures of morbidity should also be interpreted with caution given the multiple tests/models that were conducted.

### Implications

Notwithstanding the above limitations, this study is based on a national longitudinal study of community dwelling older adults (≥ 50 years) in Ireland, and enabled us to examine the reliability of two measures of morbidity across 29 conditions as well as a wide-range of potential predictors of discordance when measuring morbidity between self-report and medication-based models. The results of our study indicate that neither measure of morbidity is completely reliable. In addition, our study highlights a number of limitations to the current versions of the Rx-Risk and Rx-Risk-V medication-based measures of morbidity [9, 10]. Specifically, in the current version of the Rx-Risk and Rx-Risk-V, we identified a number of instances where commonly used medications for a condition have not been included – e.g., CCBs for hypertension; anti-epileptics for bipolar disorder; and drugs for urinary frequency and incontinence for incontinence. Further, for a number of Rx-Risk and Rx-Risk-V conditions, some of the medications identified are common across a number of conditions e.g. anti-platelet therapy is common for heart attack, stroke and mini-stroke; making it difficult to confidently establish which specific condition the patient may have.

Our study highlights the need to update the drugs included for each chronic disease class in the Rx-Risk and Rx-Risk-V in order to test the true reliability of these medication-based measures of morbidity. Indeed, if medication-based measures are to be used to assess the epidemiology of chronic conditions, then they need to be up-to-date with current evidence-based treatment guidelines, while also taking account of changing practice for the management of certain chronic conditions. This could be achieved through Delphi consensus or by review of the current Rx-Risk and Rx-Risk-V morbidities and associated prescribed medication by an expert panel. In addition, where a medication is licensed for more than one indication (e.g., amitriptyline is licensed for both depression and neuropathic pain) [61] or where the same medication or drug class is indicated for a range of conditions (e.g., anti-platelet therapy for heart attack and stroke), then the Rx-Risk and Rx-Risk-V may need to specify that patient medical data also needs to be reviewed to verify the indication for which the medication has been prescribed. Equally, as some conditions are managed using non-pharmacological means (e.g. physiotherapy for pain management), this also needs to be taken into consideration. Indeed, an integrated dispensing and medical record system, particularly across the interface between primary and secondary care, would enable a more accurate measure of medication-based morbidity [62].

There are also a number of limitations to patient self-report as a reliable measure of morbidity. Similar to the Rx-Risk and Rx-Risk-V, our findings suggest that certain morbidities (e.g. depression and emotional problems) may not be adequately measured by self-report alone; and other measures of morbidity may need to be considered alongside self-report, including physician assessment, measures that use both diagnosis codes from hospital records or medical claims and pharmacy data, [8] and/or application of validated clinical scales. More detailed and more specific questions for conditions where there are no clear diagnostic criteria and where there is variability in symptoms (e.g., incontinence, pain, COPD) may also need to be added to self-report measures, with a view to improving accuracy. There is also evidence that disease severity can influence the accuracy of self-report measures of morbidity; with previous research indicating that when severity assessment is taken into account, the predictive validity of self-report morbidity measures significantly improves in relation to health outcomes [18]. A measure of disease severity may therefore need to be included when measuring morbidity. Studies have also indicated that older patients can have insufficient disease and medication knowledge and are often not aware of what medication they are taking for their diagnosed health conditions [63]. Pharmacists and GPs in the community setting are ideally placed to provide patients with such information, which may improve the accuracy of measures of morbidity. This approach may also provide these healthcare professionals with a means of collaborative decision making and treatment planning for older patients with multimorbidity.

## Conclusion

Indeed, as our older population continues to grow, and given the significant prevalence of chronic conditions among middle-aged and older adults, researchers, healthcare professionals, and policy-makers need to be able to measure morbidity in an accurate and efficient way. To do this, a single measure which incorporates both self-report and clinical/medication-based measures, but which also captures non-pharmacological interventions used to manage chronic conditions, may therefore be necessary. A recent study comparing the measurement of multimorbidity using different data sources, has highlighted the utility in combining self-report and administrative data sources [64]. Our findings indicate the need for a similar trajectory, and we recommend that future research should employ a combination of measures in order to capture accurate prevalence estimates of multimorbidity.

## Data Availability

The data that support the findings of this study are available from Trinity College Dublin https://tilda.tcd.ie/but restrictions apply to the availability of these data and so are not publicly available.

## Appendix

**Table 5:** Conditions reported in Rx-Risk-V, but not in TILDA

	N	(%)
Epilepsy	150	(8.20)
Gout	77	(4.21)
Inflammatory Bowel Disease	23	(1.26)
Transplant	8	(0.44)
Tuberculosis	0	(0.00)
End-Stage-Renal-Disease	15	(0.82)
Allergies	340	(18.58)
Benign Prostate Hypertrophy	156	(8.52)
Hypothyroidism	198	(10.82)
Liver Failure	200	(10.93)
Migraine	2	(0.11)
Pancreatic Insufficiency	3	(0.16)
Psoriasis	24	(1.31)
Smoking Cessation	48	(2.62)
Steroid Responsive Disease^a^	429	(23.44)
Ostomy	20	(1.09)

^a^ Systemic Corticosteroid use

**Table 6:** Conditions reported in Rx-Risk, but not in TILDA

	N	%
Epilepsy	127	(11.60)
Gout	22	(2.01)
Inflammatory Bowel Disease	21	(1.92)
Transplant	6	(0.55)
Tuberculosis	1	(0.09)
ESRD	1	(0.09)
Thyroid Disorder	97	(8.86)

**Table 7:** Conditions reported in TILDA but not measured by Rx-Risk or Rx-Risk-V

	N	%
Heart Murmur	63	(2.15)
Varicose Ulcer	28	(0.96)
Serious Memory Impairment	13	(0.44)
Alzheimer’s Disease	1	(0.03)

## Abbreviations

TILDA: The irish longitudinal study on ageing
WHO: World health organisation
ATC: Anatomical therapeutic classification
ICD: International classification of diseases
MDMI: Medicines disease burden index
GP: General practitioner
ACS: Ambulatory care sensitive
HSE: Health service executive
PCRS: Primary care reimbursement service
GMS: General medical services
STROBE: STrengthening the reporting of observational studies in epidemiology
CAPI: Computer aided personal interview
CES-D: Centre for epidemiologic studies depression scale
CHF: Congestive heart failure
IHD: Ischaemic heart disease
CCB: Calcium channel blockers
COPD: Chronic obstructive pulmonary disease
OTC: Over the counter
